# Valuable unintended learning outcomes when practicum for student teachers in kindergartens is carried out online

**DOI:** 10.1007/s10639-022-11135-z

**Published:** 2022-06-10

**Authors:** Siri Sollied Madsen, Helge Habbestad, Iris H. Borch

**Affiliations:** 1 Faculty of Humanities, Social Sciences and Education, UiT the Arctic University of Norway, Tromsø, Norway; 2 Faculty of Humanities, Social Sciences and Education, UiT the Arctic University of Norway, Tromsø, Norway; 3 Centre for faculty development, Faculty of Health Sciences, UiT the Arctic University of Norway, Tromsø, Norway

**Keywords:** Practicum, Digital technology, Learning outcome, Constructive alignment, Kindergarten, Early childhood education

## Abstract

This article presents a study of an educational experiment conducted at the early childhood education programme at UiT, the Arctic University of Norway. As COVID-19 made social distancing an issue, the traditional practicum in kindergartens was moved to online platforms. Constructive alignment was used as an analytical framework to investigate the possibilities and limitations of student teachers’ learning outcomes in a collaborative online learning activity. Overall, 8 out of 9 practicum teachers found the online practicum form a better alternative than supplying the student teacher with a written assignment, and 72.7% of the student teachers agreed or somewhat agreed with facilitating learning outcomes from the online practicum regardless of social distancing. The need for professional digital competence is central, as digital technology is not simply part of pedagogical practices but is becoming an integrated part of communicating and collaborating with colleagues and parents. This study broadens the understanding of how collaborative online learning can facilitate unintended valuable learning outcomes and critically debates the limitations related to emphasising a too-strong focus on intended learning outcomes as a premise for constructive alignment in education.

## Introduction

During 2021 more than 2.6 billion people around the world were under lockdown measures due to the COVID-19 pandemic. These measures contributed to accelerate the transformation of the digital teaching mode from traditional physical teaching to online education (Maity et al., 2021). This global shift in education from traditional classroom learning to computer-based learning might, according to Lall & Singh (2020), be one of the largest “educational experiments” to date. Unlike traditional experiments that either test a hypothesis or have a scientific approach, many of the educational “experiments” due to the COVID-19 pandemic must be regarded as a quick pragmatic response to the new situation that emerged. Tomasik et al., (2021) indicated that the COVID-19 situation offers a unique natural experiment to gain a deeper understanding of learning processes before and during lockdowns by comparing traditional teaching to the increased use of online teaching.

This article presents a study of an educational activity conducted in the early childhood education programme at UiT the Arctic University of Norway (UiT). In the spring of 2020, as student teachers were about to start a three-week practicum in local kindergartens, the pandemic situation in Northern Norway was unstable. The number of COVID-19-infected citizens was rising, and the virus was detected in several schools and kindergartens. Norwegian government policy at this stage in the pandemic focused on reducing the risk of exposure to COVID-19 by social distancing and permitting as few close contacts as possible, which was especially critical for those categorised as high-risk groups and their relatives (HelseNorge, 2021). Considering the complex situation, UiT created an option for student teachers to conduct their practicum via the use of digital technology instead of physical presence in the kindergarten. As this was not previously planned for or tested, this learning activity took the form of an educational experiment.

There are some obvious limitations associated with the contextual premises regarding the original intended learning outcomes for a practicum when not physically present in the kindergarten, such as limitations related to interacting with children during the practicum. Therefore, conducting a fully online practicum is a measure to meet some of the original requirements when physical interaction between student teachers and children is not a good option or allowed due to formal restrictions. As this had never been done before, we decided to conduct a study about the outcomes and the experiences gained through this experiment (hereafter referred to as the online practicum).

There are also changes in the contextual premises, as student teachers interact with the staff through online platforms. Kaptelinin et al., (2021) highlighted a potential problem with the unprecedentedly central role of online meetings, as the pandemic severely limited the possibility of meeting physically. They addressed some concerns regarding the extensive use of online activities, including the disruption it may cause to intersubjective experiences and the participants’ thinking of themselves as a group.

In this study, we aspired to investigate the kinds of opportunities and limitations that emerged when moving a traditional practicum to online platforms. A student teacher survey and three focus group interviews with practicum teachers were conducted to gain insight into the student teachers’ and the practicum teachers’ perceptions of participating in this experiential learning activity. The student teachers were in their third year, and it was the last practicum of the study programme. The assignments during this practicum period focused on leadership of staff and managing projects aimed at developing knowledge and practice. This is in line with the formal requirements regarding kindergarten as a learning organisation (Ministry of Education and Research, 2017). During their practicum, the student teachers are assigned a practicum teacher, whose role is to guide, supervise, and assess the student teachers’ learning outcomes during this period. The practicum teacher is an in-service teacher in early childhood education, and part of the staff in the assigned kindergarten.

The empirical material was analysed and discussed using the concept of constructive alignment, in which the learning outcome descriptions, the teaching/learning activities and assessment are aligned with each other. Constructive alignment is regarded as a premise for student learning (Biggs, 1999, 2003; Biggs & Tang, 2011). By applying constructive alignment as an analytical framework for this collaborative online learning activity, we aimed to better understand how the different components in the course aligned when the traditional practicum settings were moved to online platforms. Further, we discuss how an experimental approach, not an activity predefined by the idea of constructive alignment, can foster new insights and opportunities otherwise not obtained.

### Research questions

How do student teachers and their practicum teachers perceive the learning outcomes obtained in an online practicum, and how is the constructive alignment of the course affected by moving the practicum from physical to online interaction?

## Materials and methods

This was a case study whose design was inspired by an explanatory sequential approach. A sequential approach is characterised by following up quantitative results with qualitative data (Edmonds & Kennedy, 2017). Initially, a qualitative survey was conducted to evaluate the student teachers’ perceptions of the online practicum. To gain a wider understanding of the student teachers’ feedback, we conducted qualitative focus group interviews with the associated practicum teachers.

### Student teacher survey

Within the group of 82 student teachers, 27 chose to conduct their practicums online. Of these, 22 student teachers responded to the survey, resulting in a response rate of 81.5%. The survey was conducted spring of 2021 and comprised both open and closed questions using five-point Likert-scale statements (see appendix). For all items it was also possible to select the option “I don’t know”. This option was only used by one student for one of the items. As the survey was set to require the respondent to answer all items, the overall result gave us good insight in the respondents’ position on each item. The survey was conducted using an online survey tool called Nettskjema. Nettskjema is developed by the University of Oslo and has a high degree of security and privacy (UiO, 2022). The survey was distributed during an online lecture on Zoom, on the last day of the practicum period. For those who did not attend the lecture, a link to the survey was distributed through the university’s learning management system (Canvas) directly to the students who had not attended the lecture. The results from Likert-scale statements were analysed using descriptive statistics, while open questions were categorised through thematic analysis. Open questions were important as it allows for deeper and nuanced insight. As Terry et al., (2017) claims, the researcher is never a blank slate and will bring their theoretical lens to the analysis. Since we as researchers was partially part of the process researched, the thematic analysis was characterized by being deductive oriented. Our developed theoretical concepts and theories gained through developing the learning activity “provided a foundation for ‘seeing’ the data, for what ‘meanings’ are coded, and for how codes are clustered to develop themes; it also provides the basis for interpretation of the data” (Terry et al., 2017). Acknowledging the deductive oriented processed, we also strived to inductively analyse the data bottom up, using the data as a starting point developing unforeseen themes as well as building on existing theories and expected themes.

### Focus group interviews with practicum teachers

All practicum teachers involved in the online practicum were invited to participate in focus group interviews, and 9 of 25 accepted the invitation. This resulted in three focus group interviews conducted in the summer of 2021. The focus groups varied from two to four practicum teachers per interview. The focus group discussions were based upon an interview guide (see appendix) inviting the practicum teachers to share their experiences regarding the practicum setting and how they assessed the student teachers’ learning outcomes. The interviews lasted 30–50 min and were conducted via the online platform Zoom. In agreement with the participants a function integrated in the Zoom-app was used to create a video recording of the interview. In line with Kvale & Brinkmann (2015), we understand the analysis of the material and the conduction of interview to be intertwined. When developing the interview guide, certain categories were already in mind based on the analysis of the quantitative material, and a preliminary analysis started during the interview. Subsequently, all three interviews were transcribed using the video material and word. Through this process all material was anonymised. As data from the focus group interviews was more complex than the survey data, thematic analysis of the transcribed material was conducted with the use of computer-assisted qualitative data analysis using the software program NVivo (release 1.0). The analysis involved defining themes and categories and interpreting and creating meaning based on the findings (Creswell & Poth, 2018). We conducted two independent analyses of the transcribed data and critically discussed any inconsistencies between the interpretations to strengthen the validity of the analysis.

### Ethics

The Norwegian Centre for Research Data (NSD) provides data protection services to Norwegian research and education institutions and must be notified if personal data are collected during a research project. The purpose is to ensure legal access to the necessary personal data for research. The survey conducted in this study did not collect personal information, and therefore, we did not notify the NSD. The students were informed that the survey was voluntary and conducted anonymously, and that the results could be published and used for research purposes. Based on this, informed consent was secured through oral information during a digital seminar. Those students not attending the seminar gained access to the survey through the learning management system, with written information attached.

However, the NSD was notified about the interviews, and NSD assessed that the project fell under the data protection legislation (reference number 171,230). Informed consent was secured through both written information sent by email prior to the interview, and oral information at the start of the interview. The information letter was developed and approved in accordance with the requirements from NSD, securing the participants’ right to privacy through the processing of personal data (Personal Data Act, 2018). The interview guide was also sent to the participants prior to the interviews. This was done to make the participant aware what questions we were planning to ask and to make it possible for the participants to prepare for the interview (appendix 2).

Regarding ethics and research integrity a distinction is often made between falsification, fabrication and plagiarism, and questionable research practices. While falsification, fabrication and plagiarism are found to have low prevalence, questionable research practices are claimed to be underexplored and underestimated regarding severity (Kaiser et al., 2022). Through the project Research Integrity in Norway (RINO) it is found that 40% of Norwegian researchers have committed questionable research practices during the last three years. As stated by the authors, research ethics and integrity are important for upholding trust, and for the overall quality of the scientific outputs (Kaiser et al., 2022). Therefore, it is of great importance to be critical towards ethical issues during the research process. In addition to being regulated by law (Research Ethics Act, 2017), the National Committee for Research Ethics in the Social Sciences and the Humanities (NESH) provides guidelines for research ethics (NESH, 2019). These guidelines build on the Personal Data Act and has been central in our research regarding the relationship to the students and employees who have taken part in the project, this including norms and research ethics regarding respect, human dignity, confidentiality, and free and informed consent (NESH, 2019).

## Theoretical framework: Collaborative online learning (COL) and constructive alignment

Practicums in kindergartens and theoretical lectures on campus are understood as equal arenas for learning. The handbook for practicum further defines how the student teachers are expected to interact, communicate, and collaborate with all groups in the kindergarten. Student teachers are to participate in daily tasks equal to the pedagogical leaders and early childhood teachers through joint reflection (UiT, 2020). This coincides with the notion of collaborative learning. Collaborative learning is when people share a learning task, combining expertise, knowledge and skills to improve the quality of the learning process. Collaborative learning also revolves around building or consolidating a learning community (Bernard & Rubalcava, 2000). As the third-year practicum requires the student teacher to manage and lead a group of staff during a project to improve the quality of the pedagogical work, this practicum will, to a certain extent, take form as collaborative online learning (COL). The student teachers are expected to engage the rest of the staff in their projects. Lock & Redmond (2021) writes that teacher education programmes need to provide an array of authentic learning experiences when preparing educators. This includes online learning, where learning occurs through critical discourse. Online collaborative learning should enable student teachers to integrate prior new knowledge and experiences, multiple perspectives, and higher-order thinking (Lock & Redmond, 2021).

It can be challenging when collaborative learning environments are established through digital technology and online platforms (Fung, 2004) as COL calls for social interaction. Bernard & Rubalcava (2000) indicated that COL is mostly based on the theory of social constructivism. From this perspective, when creating online learning environments, certain design issues emerge as important: (1) preparing for collaboration, (2) creating a social climate and a community of learners, (3) encouraging true collaboration, (4) applying learning approaches, and (5) using technology effectively.

This resonates with newer ideas of course design theories, such as constructive alignment. As mentioned above, obtaining constructive alignment in educational courses is achieved when there is consistency among the objectives, learning activities, and assessment exercises (Blumberg, 2009). This is an established educational theoretical concept that is used internationally. Nevertheless, Hailikari et al., (2021) claimed that the student perspective has often been neglected when exploring the concept of constructive alignment. Thus, we suggest including several perspectives when assessing this educational experiment.

Biggs (1996) understanding of constructive alignment combines two lines of thinking. It builds on constructivist learning theory, in which the students’ activities are understood as central to the learning processes, integrated with instructional design theory that emphasises the notion of the importance of alignment between objectives, learning activities, and the assessment of student performance. Inspired by Biggs’ steps to achieve constructive alignment and to provide more contextual information to the reader, we chose the following central elements as an analytical structure for this article:

1. Defining the intended learning outcomes for practicum (normally conducted physically).
2. Describing how the learning activities were redefined when planned for online platforms.
3. Assessing student teachers’ actual learning outcomes to see how well they match what was intended.

Steps one and two, defining the intended learning outcomes and describing the redefinition of the learning activities, will serve as the background and context for the study. Step three, assessing the perceived learning outcomes, is elaborated on in the sections that presents the results of the study and discussion.

## Background and context

### Intended learning outcomes for online practicums

In the curriculum, a practicum is defined as an integrated part of the course design for “leadership, collaboration and organisational development”. The third-year practicum is defined as an important arena for the learning and development of professional competencies, especially focusing on collaborative and leadership skills. During the practicum, the student teachers are supposed to participate in daily tasks in kindergarten, particularly in tasks in which they have to take responsibility as pedagogical leaders for a given group of staff. Consequently, the curriculum comprises several learning outcomes regarding the topics of leadership, collaboration, and organisational development. European educational programmes are, as part of the European educational reform described in the Bologna agreement, obliged to divide the learning outcomes into knowledge, skills, and general competencies (Sursock et al., 2010). The learning outcomes related to student teachers’ practicums are as follows:

The student teachers are expected to obtain *knowledge* about:

- Different management and organisational theories, and understandings of organisational culture as a central element in kindergarten practices.
- How different political practices affect kindergarten practices and mandates.
- Relevant research, methods, and tools as a foundation for organisational development in kindergartens, including management of children and staff.
- How management and communication affect collaborative processes with children, staff, and external public collaborative units.

Student teachers are also expected to develop *skills* regarding how to:

- Manage, plan, conduct, and evaluate a project aimed at organisational development in kindergartens.
- Supervise and critically reflect on kindergarten practices.
- Collaborate with staff, parents, and external agencies.

As *general competencies*, it is expected that student teachers:

- Gain insight into and are able to apply research-based knowledge and professional ethics in the development of ones’ role as a leader.
- Are able to manage and lead pedagogical work and make decisions that lead to the development of kindergartens as learning organisations.
- Are able to evaluate how structure and culture in and outside the organisation affect leadership and organisational development.

### Learning activity: the online practicum experiment

Before the pandemic, and as described in the curriculum, the student teachers were supposed to obtain the learning outcomes through on-campus lectures and a three-week practicum in a kindergarten. The student teachers were expected to participate in a meeting before starting the practicum and to plan the practicum and tasks together with their practicum teachers. One of the tasks related to preparing for the practicum was writing a personal development goal for the period. Each student teacher was also in charge of an assigned group of staff for five days during the practicum. They were to plan, manage, and lead a development- and/or competency-enhancing intervention aimed at strengthening the quality of the pedagogical practices. Related to this project, student teachers were to conduct supervision in groups of staff involved in the project. After the completion of the practicum, the student teachers were supposed to write a text reflecting on their own experiences of leading and collaborating through the project. When transforming this original plan of the practicum into an online practicum, the conditions for leading, managing, communicating, and collaborating with the kindergarten changed dramatically. When conducting an online practicum, all communication with the staff was through the use of technology. An evident limitation regarding the online practicum is that sufficient relations and interactions with the children, parents, and external agencies were difficult to establish. Considering previous measures in which practicums across study programmes were cancelled due to the pandemic, our intention in this experiment was to investigate whether the use of digital technology could facilitate some of the intended learning outcomes. Relevant learning outcomes were those related to project management, leadership, and organisational development.

## Results

### Learning outcomes evaluated by student teachers

After completing the three-week online practicum, 81.8% of the student teachers agreed that the online practicum contributed to developing competencies that were perceived as useful considering pandemic-related challenges. None of the student teachers positioned themselves as neutral or disagreed with this. In total, 95.5% of the student teachers agreed with the statement “I see leading meetings and supervising through online devices as an important competency for my future occupation” (77.3% agreed and 18.2% somewhat agreed). We recorded that 90.9% of the student teachers claimed that they gained competencies they would not have gained under ordinary practicum (81.8% agreed and 9.1% somewhat agreed). When investigating whether this was context dependent, only 9% disagreed with the statement that learning outcomes from online practicums should be implemented in ordinary practicums regardless of the pandemic, whereas 72.7% agreed or somewhat agreed with the suggestion regarding facilitating learning outcomes from online practicums during traditional practicums in the future.

In the open-ended question section, the student teachers were asked to supplement what they experienced as the most relevant learning outcomes. The content of their responses was categorised into five different learning outcomes, with digital meeting management and digital cooperation with kindergarten staff emerging as the most relevant learning outcomes.

**Table:** 

Learning outcomes	Number of student teachers (n = 22)
Digital meeting management and cooperation with kindergarten staff	8
Project management	5
New insight in communication	3
Seeing possibilities and adapting to changes	2
Knowledge of organisational culture and change of practice.	2
I don’t know	2
Total	22

One student teacher explained: “What has been the most relevant (learning outcome) regarding the future is that a lot of meetings will be conducted digitally and actually be led in that way”. Another said that “(a learning outcome) is to facilitate work and collaborations with the use of digital platforms. This is something I see as important in my future professional life”. Student teachers both directly and indirectly mentioned new insights into communication generated by the context. A learning outcome explicitly mentioned by three of the student teachers was their awareness of how the premises for communication changed when conducted digitally. The student teachers experienced that a higher level of clarity and precision was needed when communicating through digital technology. One student teacher explained:

The most relevant [learning outcome] must be that I have learned how clear and concise my messages need be to achieve good collaborations. Without concise information, uncertainty can arise and lead to frustration. I have also learned how important it is to include the entire group of staff throughout the process so that they can contribute and feel ownership. This is important for implementation. I have previously felt that practicum is a collaboration between the practicum teacher and the student teacher, but in this period, I have really experienced that the whole group of staff have been working together and been engaged in the project I have managed. This has resulted in good learning regarding collaboration.

The overall evaluation by the student teachers’ perspectives was mostly positive; nonetheless, they pointed out some limitations with the online practicum. Half of the student teachers expressed that not being physically present was perceived as a loss. Being present with the children was important for several of the respondents. Other expressed disadvantages due to contextual limitations regarding the possibility of obtaining instant feedback. Curtis & Lawson (2001) described the differences between collaborative behaviours in online learning and face-to-face situations. These differences include the lack of “challenge and explain” cycles of interaction that are thought to characterise good interactions. This coincides with the student teachers’ explanation of how the physical practicum better facilitates spontaneous supervision when called for.

### Learning outcomes evaluated by practicum teachers

The practicum teachers discussed the student teachers’ learning outcomes in all three focus group interviews. Eight out of the nine practicum teachers claimed that the online practicum served as a better alternative compared to assigning student teachers with written assignments detached from kindergartens and their practicum teachers during pandemics. The practicum teachers explained that even though there were obvious limitations, the student teachers were generally assessed to have acquired important experience through establishing a digital connection with the field of practice. Seven of the practicum teachers mentioned that the student teachers gained experience of conducting and leading an online meeting, and that they saw it as a central learning outcome. Even though the technical aspects of using the online platform were within the student teachers’ existing competencies, the practicum teachers explained how the learning activities resulted in deeper reflections and discussions regarding the implications of moving a professional meeting to an online platform. Several practicum teachers also mentioned that merely becoming comfortable conducting online meetings was perceived as an important learning outcome in itself, for both themselves and the student teacher. The increasing need to develop kindergarten teachers’ professional digital competence is an issue addressed by several practicum teachers. One described that digital competency would be useful for all employees in kindergarten, explaining that:

One has to be comfortable with communicating digitally, and not to feel discomfort when participating in an online meeting. It is good to have the opportunity to meet, even when you are not at the same location. During our practicum, we experienced how to establish a reflective practice together with our peers/colleagues. To me, this has been a positive experience.

Another practicum teacher said:

Both my student teacher and I concluded that it was a good experience regarding how to lead digital meetings involving both colleagues and parents. When in lockdown, this was how we worked. It is important to be comfortable with the technology and how it can solve different challenges. Acquiring different methods to conduct your job when one cannot meet physically is important. Then, one does not need to put everything on hold when events such as sick leave happen. I believe times are changing and conducting meetings will be more digitised in future practices.

Being comfortable with the tool is important, but acquiring knowledge of how the context is setting premises for communication is also important. Six of the practicum teachers emphasised a central learning outcome related to gaining a broader understanding of the different premises for communication when conducted through digital platforms. One practicum teacher described how they gained a broader understanding of the difference between written and oral communication, while others focused on the understanding gained regarding the difference between physical presence and interacting through a screen. Both the supervision experience and the communication processes when leading the student teachers’ projects were perceived as important parts of the third-year practicum.

When it came to assessing the student two of the practicum teachers discussed a discomfort related to the lack of physical interaction when practicum was carried out online. One practicum teacher explained:

I really struggled to write that assessment because I really did not have anything to say. I could assess her based on her written and didactical work… and I could assess her based on how we communicated… that is actually a big part of what we are engaged in (reflecting on his own statement).

Another explained, “The foundation for assessment bothered me a bit. First and foremost because one does not get to see how the student teachers are interacting with the children, and that… that makes me uncomfortable. A feeling of unease”.

## Discussion

### Comparing student teachers’ achieved learning outcomes with intended learning outcomes

Related to functioning as the pedagogical leader for a given group of staff, the student teacher gained experience and several of the intended learning outcomes were achieved through the online practicum. The student teacher gained experience with and knowledge of how management and communication affect collaborative processes with staff. Student teachers were able to test relevant methods and tools as a foundation for managing staff and organisational development in kindergartens. The pandemic situation offered the student teachers insight into how different political practices can affect kindergarten practices and mandates. The student teachers also developed skills regarding how to manage, plan, conduct, and evaluate a project aimed at organisational development in kindergartens. They supervised staff and were involved in critically reflecting on kindergarten practices. Through this process, they had to collaborate with the staff to make an impact on the children in their project.

### Limitations and premises for learning outcomes

During an online practicum, the student teacher is limited to participating in only some of the daily tasks in the kindergarten, compared to being physically available during the practicum. This affects their ability to achieve learning outcomes related to physical interactions with children, parents, and external agencies. In our study, student teachers who conducted the online practicum were vocal regarding the importance of the practicum teacher being positive towards using digital platforms as a medium to conduct the assignment. This was also reflected in the survey results. As Thorpe (2002) explained, in COL activities, substance and meaning are determined by those who work together online. Having their practicum teacher play a very direct role in helping during their projects shapes these interactions. Supporting and preparing for interpersonal connectedness, “team experience”, and the sense of being “we” in online meetings is central (Kaptelinin et al., 2021).

### Lack of constructive alignment when assessing

When practicum is conducted, student teachers’ learning outcomes are assessed by the practicum teachers to determine how well and to what degree they match the intended learning outcomes. When moving traditional learning concepts to online platforms, it is important to prepare the participants to adapt their expectations according to changes in context. Prins et al., (2005) indicated that computer-supported collaborative learning is not merely a matter of changing the technology. It requires a redesign of the learning environment, which includes assessments. When the learning activity was not simply computer-supported but exclusively online, the need to redesign assessment became evident. Some of the practicum teachers found it hard to assess the student teachers, as there was a missing link between the online learning activity and how the traditional assessment was conducted. As it was uncertain what learning outcomes to expect, no changes were made to the framework for evaluation related to practicum at UiT. As students’ actual learning outcomes turned out to be mainly related to digital leadership and communication, the original document guiding the assessment of students did not match the conducted learning activity. For two of the practicum teachers, there seemed to be a reluctance regarding looking beyond the limitations of what traditional practicum entails to how online practicums enable potential learning outcomes.

One of the practicum teachers’ scepticisms about online practicums was concerning the student teachers’ limited opportunities to develop interpersonal qualities. Interpersonal qualities are professional aspects in early childhood education that call for training and physical interaction with the kindergarten, including the children, parents, and staff. Our goal is to contribute to developing knowledge of how learning institutions can facilitate professionally relevant learning when physical interaction is not an option. Our study also indicates that developing student teachers’ digital competence is relevant for their future profession in kindergartens and should to some degree be integrated as an intended learning outcome during their physical practicum period to make them prepared for the future.

### Unintended but valuable learning outcomes

Loughlin et al., (2021, p. 130) offered a criticism of constructivism by prescribing that “fixed outcomes cannot represent the unanticipated consequences of teaching”. Unintended and valuable learning outcomes that were initially not part of the intended learning outcomes were student teachers gained knowledge of and competencies regarding digital leadership, digital communication, and new practices related to digital collaboration with colleagues, parents, and external public units. A common misunderstanding is that the younger generations, also referred to as digital natives when born after 1980 (Prensky, 2001), naturally and intuitively adapt to digital technology as an integrated part of society (Flynn, 2021; Terry, 2018). Based on our own experiences with online teaching during the pandemic, being comfortable with interacting through online platforms seems to be a challenge across contexts. This has also been highlighted in research, where students across nations and subjects are often reluctant to participate digitally, often referred to as the black screen phenomenon (Christoforou, 2021). This indicates a need to not overestimate the younger generation’s ability to convert their existing digital skills to a more complex understanding of professional digital competence in early childhood education (Casillas Martín et al., 2020).

The design of computer-supported collaborative learning is, according to Kirschner et al., (2004), probabilistic. The authors posited that designers of education often think they know what the designs will do and how students will use them. In line with our experience with online practicums, they claim that this is not always the case. Kaptelinin et al., (2021) indicated that “the current massive transition from physical to online meetings may mean that complete “team lifecycles”, from emergence to dissipation, will increasingly involve little or no physical meetings”. This trend is also evident within kindergarten education, as the use of online meetings is becoming a part of normal practice. Based on this, we question whether it is constructive for educational development to always know what the learning design will do, or which learning outcomes it will result in. The level of detail in intended learning outcomes will affect the participants’ ability to innovate, reflect, and contribute to individual solutions. Entrepreneurial teaching is often characterised by the fact that there is no clear or unambiguous connection between intended learning outcomes and learning activities (Andersen, 2010).

## Innovative possibilities can emerge from a lack of constructive alignment

This article builds on the idea of constructive alignment (Biggs & Tang, 2011), but the experience and reflections made during the process of online practicum calls for a critical discussion regarding how to understand constructive alignment in higher education. Regarding the pandemic, Kim (2020, p. 153) claimed that the situation calls for appropriate and efficient ways to develop student teachers’ skills:

Frustrations emerged from uncertainty about what and how to deal with the circumstances that educators or student teachers had not experienced before. However, whether it is online or in person, teacher educators are required to find appropriate and efficient ways to help develop the skills of their students for problem-solving.

Education has internationally been inspired by constructive alignment (Andersen, 2010; Gynnild et al., 2021; Loughlin et al., 2021), and is described as one of the most influential ideas in higher education (Houghton, 2004). What is often understood as appropriate and efficient from this perspective is the quality of predefined course alignment. Nevertheless, the concept has been widely debated. Andersen (2010) claimed that when constructive alignment is the foundation for developing education, a risk regarding generalisation and simplification follows. Her claim is that a rigid implementation of the concept hinders deep understanding and creativity. Andersen’s critique could be understood in light of Loughlin et al., (2021) descriptions of how constructive alignment has developed from theory to policy and practice. This development has created an illusion of systemic integrity at odds with reality in higher education. Constructive alignment has been drawn under the umbrella term “outcome-based education”; however, Loughlin et al., (2021, p. 120) argued that constructive alignment is a misinterpreted qualitative tool, “whose success in practice is predicated on implementation by skilled, professional educators”. They argued that reclaiming the original intention of the theory of constructive alignment is needed if higher education is serious regarding student-centred learning. As Biggs described in his earlier work, “to make the objectives upfront and salient is not to exclude other desirable but unforeseen or unforeseeable outcomes” (Biggs, 1999, p. 43). As this study was a measure in times of crisis, some of the practicum teachers felt unprepared when sudden changes in concepts emerged. Even though it was a sudden change in context, it seemed that the student teacher and practicum teacher engaged in creating a social climate for learning. To what degree the online practicum encouraged true collaboration is hard to say, but, as both student teachers and practicum teachers suggested, this form of practicum can be considered an improvement of existing pandemic-related practices.

## Conclusions

As the learning activity during the student teachers’ practicums changed from being based on physical presence to online interaction, the premises for the intended learning outcomes changed accordingly. This resulted in several achieved learning outcomes that were expected, but also some unintended learning outcomes. The achieved intended learning outcomes were outcomes such as gaining experience with managing a group of staff and implementing a joint project to develop practice. In addition to the expected learning outcomes, several valuable unexpected learning outcomes were mentioned as well. Such as developing the professional digital competence needed to manage staff when interacting online and gaining knowledge of how the digital context is affecting the premises for communication. The extensive use of digital technology was described as a steep learning process for both the student teachers and the practicum teachers, but the need for professional digital competence in kindergarten was understood as becoming an integrated part of kindergarten practices when communicating and collaborating with both colleagues and parents.

In times of social distancing this form of practicum was perceived as a better option, compared to previous written assignments as a substitute for practicum. Researchers have also concluded on the value of social learning activities in education, and Kirschner et al., (2004) indicated that higher education suffers from stress on the individual acquisition of knowledge and skills. Following this notion, they questioned the validity of traditional education. This is explained by the fact that certain learning goals are difficult to achieve in an individual context. They exemplify this with negotiation skills, chairing a meeting, monitoring, and discussing. Based on this knowledge and our findings from this experiment, it is crucial to be aware the need for social learning activities when educating student teachers. This is especially important during times when social distancing is needed due to pandemics. We, as educators, need to develop alternative ways to ensure collaborative learning when external issues are pressing for isolation and individual learning activities. Conducting an online practicum is not ideal as several important elements are lost in the transformation of context. Nevertheless, working to achieve the learning outcomes gained through social learning seems to be constructive and valuable for student teachers.

According to Kim (2020), generating alternatives is the means of modifying the original plan in the best ways possible, and sometimes the alternative way becomes the better way of doing things. The alignment of the learning activity was apparently weakened by moving the practicum from physical to online interaction, but when accepting the apparent lack of alignment unforeseen possibilities emerged. The findings from this study have broadened the understanding of how COL can facilitate learning outcomes previously related to traditional practicums while critically debating the limitations related to emphasising a too-strong focus on intended learning outcomes and constructive alignment as a theoretical premise for quality in education.

## Data Availability

The datasets generated by survey during and analysed during the current study are available from the corresponding author on reasonable request. Dataset generated through interviews during and analysed during the current study are available as edited version from the corresponding author on reasonable request, this due to securing the participants anonymity.

## Appendix 1: Student survey

Likert scale statements were prepared with a five-point scale (1. agree, 2. somewhat agree, 3. neutral, 4. somewhat disagree and 5. disagree). In addition to this the option “I don’t know” was also an option.

- Online practicum has contributed to the development of competencies that can be valuable for pandemic-related challenges.
- There are working methods and content in online practicum that should be implemented in future practicum periods for future student teachers in the kindergarten teacher education.
- Online practicum did not contribute to learning on my part.
- I consider gaining experience with meeting management and online supervision as an important competence for my future profession.
- Online practicum is wanted in situations that challenge the conduction of ordinary practice.
- I experience having more time to read literature and to be more prepared for assignments during online practicum.
- I experience that the structure of online practicum better facilitates constructive use of time, compared to physical practice.
- Online practicum has led to experiences and competencies that I would not have gained through ordinary practice.
- It is important for the implementation of online practicum that the practicum teacher is positive towards this form of practice implementation.
- The practicum teacher has been critical of my choice of online practicum.
- I find it challenging to be active in professional forums with the use of digital technology.
- I would recommend others in a pandemic situation to choose online practicum.
- A selection of learning outcomes gained through online practicum should be incorporated in ordinary practice, also under normal circumstances (without social distancing).
- I have acquired skills that I would not have gained through ordinary practice.
- The practicum seminars have been useful during online practicum.

**Open-ended questions**:

- Describe how you experienced online practicum.
- What benefit did you get from the practicum seminars arranged by UiT?
- What support for conducting an online practicum was (or would have been) essential for your learning outcome?
- What have you learned that has been the most relevant for you during the period of online practicum?
- Have you gained experience with online practicum that can be valuable for UiT to build on when future practices are to be planned and conducted?
- What has been the biggest challenges during online practicum?
- Are there important elements from physical practicum that you feel you have lost? If so, which ones?
- What has been the most educational?
- What could have been done differently, and how?
- Have you acquired competencies that you would not have acquired through physical practicum? If so, describe which ones.
- What would an ideal alternative to physical practicum look like if you were to define it?
- Are there other things related to online practicum that you think will be useful for us to know about not covered in this form?

## Appendix 2: Interview guide for practicum teachers

- What was your immediate reaction to the measures prior to this year’s practice?
- How did you experience being a practicum teacher during the online practicum?
- What kind of experience did you gain through online practicum?
- What learning outcomes did you experience the student achieved during the period?
- What limitations occurred when conducting online practicum?
- What should have been done differently, and how?
- Are there elements from online practicum that should be integrated into the education of kindergarten teachers, regardless of pandemic?
- Is this form of practicum preferable in circumstances where physical participation is not possible?
